# Mechanoresponsive regulation of fibroblast-to-myofibroblast transition in three-dimensional tissue analogues: mechanical strain amplitude dependency of fibrosis

**DOI:** 10.1038/s41598-022-20383-5

**Published:** 2022-10-07

**Authors:** Diego Jacho, Agustin Rabino, Rafael Garcia-Mata, Eda Yildirim-Ayan

**Affiliations:** 1 Department of Bioengineering, University of Toledo, Toledo, OH USA; 2 Department of Biological Sciences, University of Toledo, Toledo, OH USA

**Keywords:** Regenerative medicine, Tissue engineering

## Abstract

The spatiotemporal interaction and constant iterative feedback between fibroblasts, extracellular matrix, and environmental cues are central for investigating the fibroblast-induced musculoskeletal tissue regeneration and fibroblast-to-myofibroblast transition (FMT). In this study, we created a fibroblast-laden 3D tissue analogue to study (1) how mechanical loading exerted on three-dimensional (3D) tissues affected the residing fibroblast phenotype and (2) to identify the ideal mechanical strain amplitude for promoting tissue regeneration without initiating myofibroblast differentiation. We applied uniaxial tensile strain (0, 4, 8, and 12%) to the cell-laden 3D tissue analogues to understand the interrelation between the degree of applied mechanical loading amplitudes and FMT. Our data demonstrated that 4% mechanical strain created an anabolic effect toward tissue regeneration, but higher strain amplitudes over-stimulated the cells and initiated fibrotic tissue formation. Under increased mechanical strain amplitudes, fibroblasts were activated from a homeostatic state to a proto-myofibroblast state which resulted in increased cellularity accompanied by increased expressions of extracellular matrix (ECM) components, activation stressors (TGF-β1 and TGF-βR1), and profibrotic markers. This further transformed fibroblasts into α-smooth muscle actin expressing myofibroblasts. Understanding the interplay between the applied degree of mechanical loading exerted on 3D tissues and residing fibroblast phenotypic response is important to identify specific mechanomodulatory approaches for tissue regeneration and the informed mechanotherapy-guided tissue healing strategies.

## Introduction

Musculoskeletal tissue injuries account for 65% of all occupational-related injuries in the United States and have become the fastest-growing injury type^1^. Prior to surgical intervention, non-steroidal anti-inflammatory drugs (NSAIDs)^2,3^ and physical therapy^4–6^ are commonly used as initial treatment options in clinical settings for many musculoskeletal injuries, which often fail to repair and restore the native tissue function. The musculoskeletal tissue repair process can have two distinct paths: regenerative path and fibroplasia or fibrosis path^7^. In the regenerative path, the cells at the injury site differentiate or are replaced by the cells with the same phenotype for normal parenchymal tissue. In the fibrosis path, the pathogenic repair process is initiated by the phenotypic changes of cells at the injury site where cells continuously secrete fibrogenic cytokines and deposit an excessive amount of extracellular matrix (ECM) components such as collagen and fibronectin which further turn into permanent scar tissue^8,9^. The fibrotic tissue with inferior properties such as lower mechanical strength with lower load transmitting capacity compared to normal tissue inhibit musculoskeletal tissue functions and restricts locomotion.

Fibroblast-to-myofibroblast transition (FMT) is a key cellular mediator in the development of fibrotic tissue during tissue healing and regeneration processes. Considering the fact that fibroblasts are often utilized in numerous musculoskeletal tissue regeneration applications, efforts have been put into creating models to understand the factors modulating the homeostatic fibroblasts’ phenotypes and push them towards myofibroblast differentiation. The wound healing response includes different intracellular and extracellular events. These events initiate from inflammation, epithelialization, cell proliferation, and cell migration to fibroblast proliferation, collagen synthesis, wound contraction, and finally tissue remodeling. During the last phase of tissue remodeling, a systematic degradation of granulation tissue occurs and is replaced with a more organized and elastic ECM. This process may be induced by the secretion of matrix metalloproteinases (MMPs) during mechanical stimulation^10,11^. Furthermore, mechanical stimulus directly applied to the site of injury has been shown to facilitate scar healing by decreasing blood flow and edema and increasing collagen turnover and ECM remodeling^12^.

The reductionist two-dimensional (2D) membrane models, in which mechanical strain is applied to a fibroblast monolayer have been instrumental in understanding the mechano-responsiveness of FMT^13–17^. Additionally, 2D in vitro models studying the role of mechanical strain and stiffness on cell behavior provides the ability to quantify traction forces using time-lapse microscopy. However, these models fail to accurately mimic the interstitial feedback conditions from the surrounding ECM and adhesion fields experienced by the cells in vivo^18^. Moreover, fibroblast culture in 2D models tends to resemble a pancake shape, spreading to cellular extensions and constraining its migration as compared to the spindle or stellate shape as observed in 3D or in vivo models. Thus, failing to accurately replicate cell migration, adhesion, proliferation, and further differentiation response to mechanical forces compared to models grown in 3D tissue analogues^19^.

Currently, 3D floated cell-encapsulated collagen lattices are the most utilized in vitro model to understand the interaction between fibroblast cells and the ECM and whether this interaction promotes myofibroblast differentiation^20–22^. In the collagen lattice model, cells within the collagen create small tractional forces during the attachment and migration within the matrix which leads to a reduction in floated collagen lattice diameter^20–22^. Besides collagen lattice models, other prominent studies created a 3D matrix model to investigate the role of changing ECM mechanical stiffness on fibrotic tissue formation and myofibroblast differentiation^23–26^. Although these models enable fibroblast cells to interact with 3D ECM to study FMT, they ignore the extracellular mechanical loading exerted on the ECM and the residing cells. Yet, in clinical settings, the importance of extracellular mechanical loading on fibrotic tissue formation has been recognized for fibrosis-associated disorders. Mechanical loading-induced reorientation of the wound and pressure therapy are commonly used in the treatment of fibrotic tissue formation^10,27,28^. Thus, understanding the pathophysiology of mechanical loading mediated fibroblast-to-myoblast transition is crucial for creating efficient therapies for musculoskeletal tissues operating under constant mechanical loading conditions. There remains an unanswered question regarding how the degree of mechanical loading applied to a fibroblast-laden 3D matrix and associated changes in structural and mechanical properties of the matrix affect the shift from normal tissue repair to fibroblast-to-myofibroblast transition.

To this end, in this study, our objective was to investigate the dose–response relationship between various mechanical strain amplitudes applied to a 3D tissue analogue, subsequent structural changes within the tissue, and the molecular changes in residing fibroblasts that regulate the fibroblast transition from the homeostatic state to differentiated myofibroblasts.

## Methods and materials

### Synthesizing of fibroblast-laden three-dimensional tissue analogue and mechanical loading

The three-dimensional (3D) tissue analogue material was prepared using the most important structural extracellular matrix proteins of collagen and elastin. The elastin component of the tissue was prepared by dissolving 375 mg of human soluble elastin (ES12, Elastin Production USA) in 75 mL Tris Buffer 1 M at a pH of 7.6 and then incubating it in a shaker water bath at 37 °C for 24 h. The functionalized elastin solution was further diluted to 0.4 mg/mL to admix with 4 mg/mL neutralized collagen solution. The neutralized collagen solution was prepared from 8.9 mg/mL collagen Type-I solution (Corning, USA) with a pH of ~ 3.4 through mixing with 1 M sodium hydroxide, phosphate buffer solution (PBS), and deionized water using our well-established protocols^29–31^. Then, the human dermal fibroblasts (ATCC, HFF1; USA) were cultured in a complete media of Dulbecco’s modified Eagle’s medium (DMEM) (ATCC; USA) supplemented with 15% fetal bovine serum (FBS) (Corning; USA), and 1% penicillin–streptomycin (Corning; USA) were encapsulated within the composite biomaterial with a cell density of 10^6^ cells/mL.

**Figure 1:**
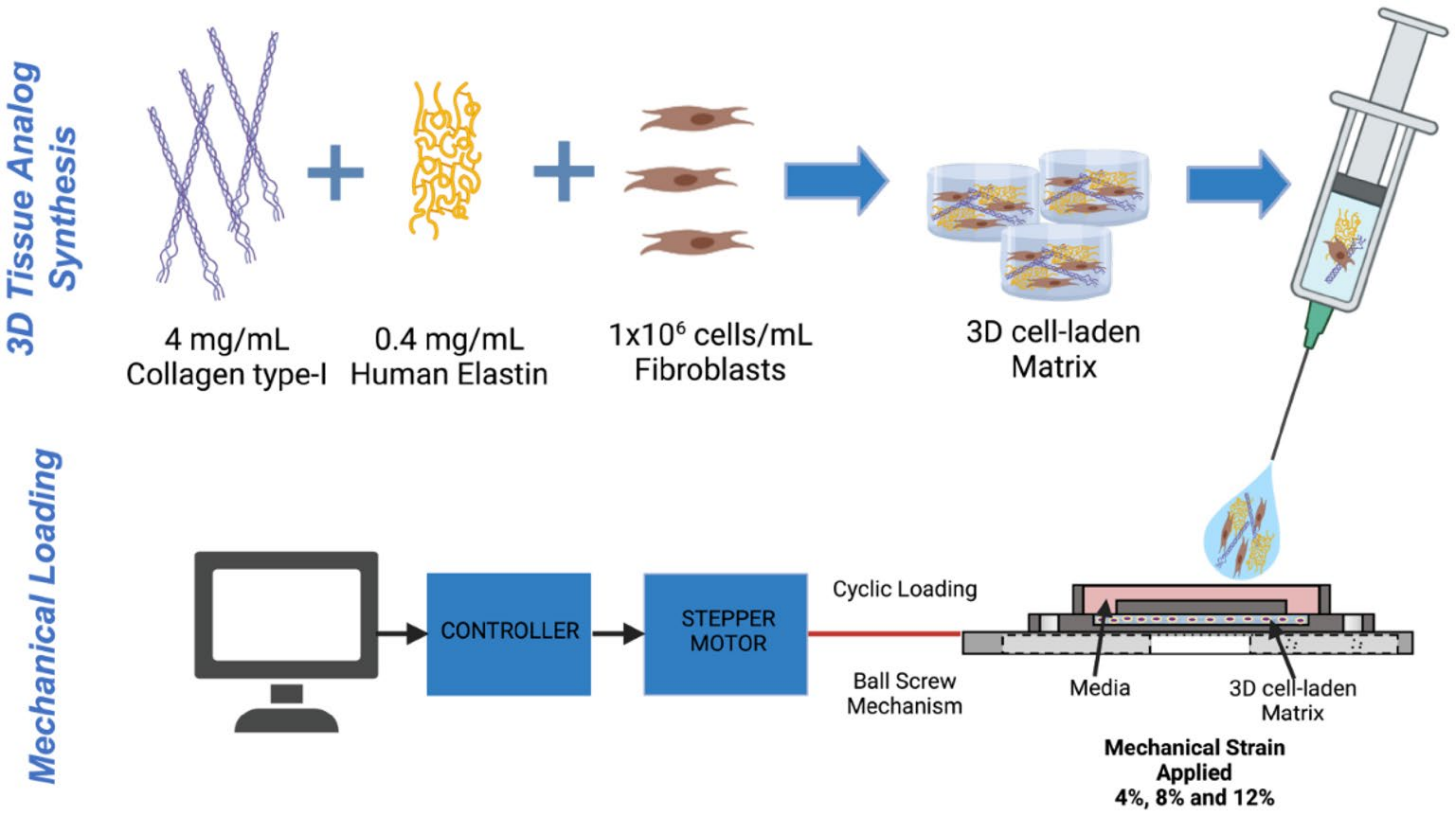
Schematic representation of cell-laden 3D tissue analogue synthesis and mechanical loading platform of cell-laden construct. “Created with www.Biorender.com”.

The fibroblast-laden 3D tissue analogue was then deposited inside the mechanical loading chamber and incubated at 37 °C and 5% CO_2_ for 2 h for polymerization. After polymerization, 1 mL of complete media was added to the loading chambers and incubated for 24 h before the mechanical loading. The next day, fibroblast-laden tissue analogues were exposed to various uniaxial tensile strains (4, 8, and 12%) with 0.1 Hz frequency using a custom-built validated Uniaxial Strain Bioreactor^31,32^. The 4%, 8%, and 12% uniaxial tensile strain at 0.1 Hz frequency was applied to the tissue analogues to mimic the dynamic physiological environment of musculoskeletal tissues such as tendon, ligament, and muscle for 2 h per day throughout the 7-day culture period. The different uniaxial tensile strains represent the stress experienced by skeletal tissues, reaching as much as 12.5 times body weight during exercise, a 5–9% change in length during moderate physical activities, and up to 12% during high-intensity activity^12,33–36^. Unstrained (0%) cell-laden tissue analogue was used as the control group. After 7 days of mechanical loading, the 3D tissue analogues were harvested for characterization assays, including cell viability, matrix organization, and gene expression profiles. Figure 1 illustrates the steps followed during the cell-laden 3D tissue synthesis and mechanical loading.

### Assessing the cellularity and fibroblast morphology within 3D tissue analogue with mechanical loading

The presence of cells within the 3D tissue analogue was assessed using Calcein-AM and ethidium homodimer-1 Live-Dead Assay (Life Technologies, USA). On characterization day, the samples were washed with dPBS and incubated for 2 h with a 1: 4 ratio of calcein-AM and ethidium homodimer-1 dyes in DMEM at 37 °C. Subsequently, the samples were fixed with 4% paraformaldehyde (Sigma, US) for 30 min at RT and washed with dPBS and 0.2% Tween (ThermoFisher. USA) solution. The samples were then mounted on a u-slide 8-well glass bottom (Ibidi, USA) with 200 μL of mounting media (Paramount, USA) for confocal imaging.

Samples were imaged using a 63× oil, 1.4 NA or 20× dry, 0.75 NA objective with an Andor Dragonfly 200 spinning disk Confocal Microscope mounted to a Leica DMi8 microscope and Fusion software. Image acquisition was made at 490/545 nm and 590/685 nm excitation/emission wavelengths to visualize live (green) and dead (red) cells, respectively. For quantitative analysis, at least three fields of view per condition from each biological replicate were analyzed using ImageJ (n = 3). The acquired z-stacks corresponding to 50 µm (stack size = 600 nm) were processed for background removal and quantification. In brief, to account for the loss of signal due to penetrance of the laser in the 3D analogues, the 50 um stack was divided into two subgroups, and background removal was carried out relative to the signal in each subgroup. Particle analysis was carried out in each channel, and live/dead quantification was expressed as a percentage of total cells in the field of view. Lastly, the total number of cells in each z-stack was used to extrapolate the number of cells/mL in a sample. Cell viability quantification was expressed as a percentage of live cells per total number of cells for each sample.

The effects of uniaxial loading on the fibroblast morphology were assessed by the amount of elongation of the cells within the 3D tissue analogues. The cell elongation index (EI) was obtained as the ratio of the long axis to the short axis of the cell, based on a 3D rendering of serial optical slices collected by confocal microscopy stained with F-actin. Individual cells within each image were analyzed using Fiji/ImageJ (NIH, USA) software. A cell elongation index of 1.0 represents a spheroidal cell, while an elongation index greater than 2.0 represents an elongated cell. For this analysis, three samples per experimental group were analyzed, with n = 25 cells per sample.

### Changes in matrix remodeling within 3D tissue analogue with mechanical loading

The changes in the fibrous proteins’ density (collagen and elastin) and porosity of 3D tissue analogue with various mechanical loading magnitudes were examined using Scanning Electron Microscopy (SEM) (Hitachi, USA). Briefly, on characterization day, the 3D analogues were cut in the sagittal plane and fixed with 4% paraformaldehyde in PBS for 30 min. After fixation, the samples were dehydrated first in sequential ethanol solutions with increasing concentrations from 30 to 100% for 15 min each. Dehydration was then continued by submerging samples into sequential ethanol/hexamethyldisilane (HMDS) solutions from 30 to 100% for 10 min each for image quality enhancement. The samples were then air-dried overnight. The dried samples were then gold-sputter coated and imaged vertically (parallel) to the direction of the uniaxial tensile strain applied. For matrix porosity quantification, the SEM images (n = 3) were processed using Fiji/Image J (NIH, US). The ND plugin calculated the total area of pores and fibers in the matrices^37^. The threshold was adjusted to a level of measurement up to the grey density of the collagen and elastin fibers^38^. Then, % porosity was calculated by the ratio between total area and area of pores present in the matrix. The collagen fiber alignment was assessed using the SEM images. The directionality of the collagen fibers was plotted and calculated by directionality histograms (n = 3) using Fiji/ImageJ Directionality plugin (NIH, US)^32^.

### Changes in cytoskeletal component and of residing cells within 3D tissue analogue upon mechanical loading

F-actin and α-SMA changes in cells residing in the 3D tissue analogues were evaluated by immunohistochemistry (IHC) after the mechanical loading. On characterization day, the 3D tissue analogues exposed to 0% (control), 4%, 8%, and 12% mechanical strains were fixed in 10% paraformaldehyde, dehydrated using an ethanol gradient, and cleared with xylene before being embedded in paraffin. Then, paraffin-embedded samples were sectioned into ~ 40 μm thick slices using a microtome, mounted on glass slides, and cleared using a xylene/ethanol rehydration protocol before staining.

For immunostaining, samples were permeabilized with 0.1% Triton x-100 in PBS. Then, sections were washed and blocked in 2.5% goat serum and 0.2% Tween in PBS for 20 min, followed by 20 min blocking in 0.4% BSA and 0.2% Tween in PBS. After blocking, slides were incubated overnight at 4 °C in mouse anti-Smooth Muscle Actin (1:50) (AB262054, Santa Cruz). The next day, slides were washed with PBS and 0.2% Tween solution and blocked in 0.4% BSA and 0.2% Tween solution for 20 min at RT. After blocking, samples were incubated for 2–3 h at RT with Alexa fluor 594-Goat anti-mouse IgG (1:50) (A32730, ThermoFisher, USA) and Alexa Flour 488 Phalloidin (1:50) (ThermoFisher, A12379). Hoechst dye (1:100) was used to stain for nuclei. Finally, samples were washed with 0.2% Tween and mounted in Permount mounting media (Permount, USA) with glass cover slides. The samples were left in the dark overnight at 4 °C before imaging.

Slides were imaged using a 63× oil or 20× airy objective with a Leica Stellaris 5 confocal system equipped with HyD detectors and the LASX software. The acquired images were then post-processed and analyzed using ImageJ. Briefly, confocal images from at least three fields of view from each experiment were processed to measure F-actin intensity from each cell. The segmentation of cells was achieved using both signals (Hoechst and F-actin), and the mask obtained was used to measure F-actin intensity. The integrated density values obtained were then corrected by subtracting the background signal.

### Changes in molecular blueprint of the residing cells within 3D tissue analogue upon mechanical loading

To understand the changes in profibrotic and extracellular matrix markers with increased mechanical strain amplitudes, the gene expression analysis was carried out using a quantitative real-time polymerase chain reaction (RT-qPCR). Briefly, on characterization day, the 3D tissue analogues were mechanically disrupted, then the RNA was extracted using TRIzol reagent (ThermoFisher, USA). The isolated RNA was reverse transcribed to cDNA using Superscript IV kit (Invitrogen, USA) per the manufacturer’s protocols. RT-PCR was performed using TaqMan SYBR (Bio-Rad, USA) in the iCycler iQ detection system (Bio-Rad, USA). The relative gene expression for fold difference between strained samples and non-loaded control samples was obtained using the ∆∆Ct method. In the ∆∆Ct method, Glyceraldehyde-3-phosphate dehydrogenase (GAPDH) was used as the housekeeping normalizing gene. The primer sequences for each gene were designed using NCBI primer blast software and synthesized by Integrated DNA Technologies (IDT, USA). The list of genes and associated primer sequences are listed in Supplementary Table 1 and provided as a Supplementary Document.

### Statistics

Statistical analysis was conducted using RStudio. Statistical significance was analyzed using one-way analysis of variance (ANOVA) and post-hoc analysis (Tukey test) or Student’s t-test where appropriate. The data is reported as the mean ± standard deviation. The sample size is indicated within the corresponding figures. Asterisks (*) indicate a significant difference with respect to the 0% mechanical strain group (control). Pound signs (#) represent a significant difference between 4 and 8% mechanical strain groups, § represent a significant difference between 8 and 12% mechanical strain groups, and crosses (†) represent a significant difference between 4 and 12% mechanical strain groups, each with *P* < 0.05.

## Results

### Hyper-mechanical strain increased cellularity within 3D tissue analogues

**Figure 2:**
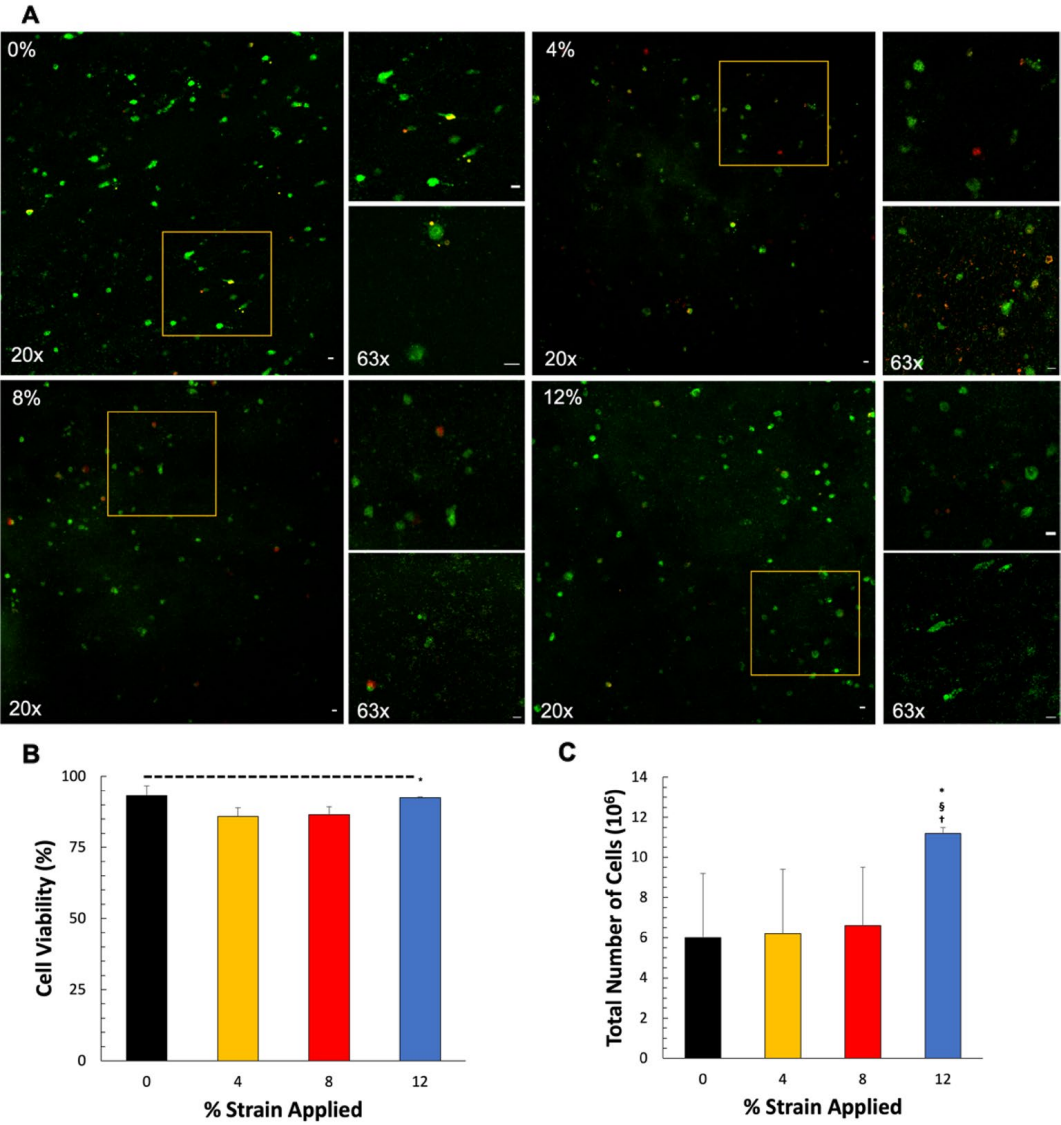
Mechanical loading increases cellularity within 3D tissue analogues. (**A**) Live/dead images of residing cells within 0%, 4%, 8%, and 12% mechanical strain 3D tissue analogues. (**B**) Percentage cell viability within 3D tissue analogue, and (**C**) total number of cells within 3D tissue analogue. Live cells were stained with Calcein AM (green). The scale bar represents 10 μm (n = 3). *Indicates a significant difference with respect to the 0% strain group (control). ^§^Represents a significant difference between 8 and 12% mechanical strain groups, and ^†^represents a significant difference between 4 and 12% mechanical strain groups, each with P < 0.05.

3D tissue analogues were prepared using rat-tail type I collagen and human elastin and seeded with human dermal fibroblasts at 10^6^ cells/mL density. After 1 day of culture, 3D analogues were exposed to uniaxial tensile loading of 4%, 8%, and 12% strain at 0.1 Hz frequency for 2 h/day for 7 days. The 3D tissue analogue exposed to various mechanical strain amplitudes was analyzed to assess changes in cellularity. As seen in Fig. 2, the number of live cells increases according to the mechanical strain, 12% being the most drastic increase (Fig. 2A). Then, we extrapolated the number of cells per ml, and the results are shown in Fig. 2C. The results indicate that 12% mechanical strain increased the number of cells per ml by almost twofold (12 × 10^6^ ± 1.78) compared to the 3D tissue analogue exposed to 0, 4, and 8% mechanical strain. There was no significant difference observed between cell numbers for the tissues exposed to 0, 4, and 8% mechanical strain. Figure 2B shows the percentage of cell viability of all groups after mechanical stimulation. 4% and 8% groups show an approx. 85% cell viability, while 0% and 12% show an approx. 92% cell viability. Additionally, looking at the morphology of fibroblasts subjected to mechanical loading, a prominent change is evident in the sample loaded with 12% strain at 0.1 Hz frequency, where the cells are elongated, appear to have spindle-shaped cytoplasmic extensions, and are in the process of orienting themselves within the matrix (Fig. 2, 63×). Cells from the other groups have a rounded appearance like those of the nonloaded control samples.

### Hyper-mechanical strain reduced tissue porosity of 3D tissue analogues

**Figure 3:**
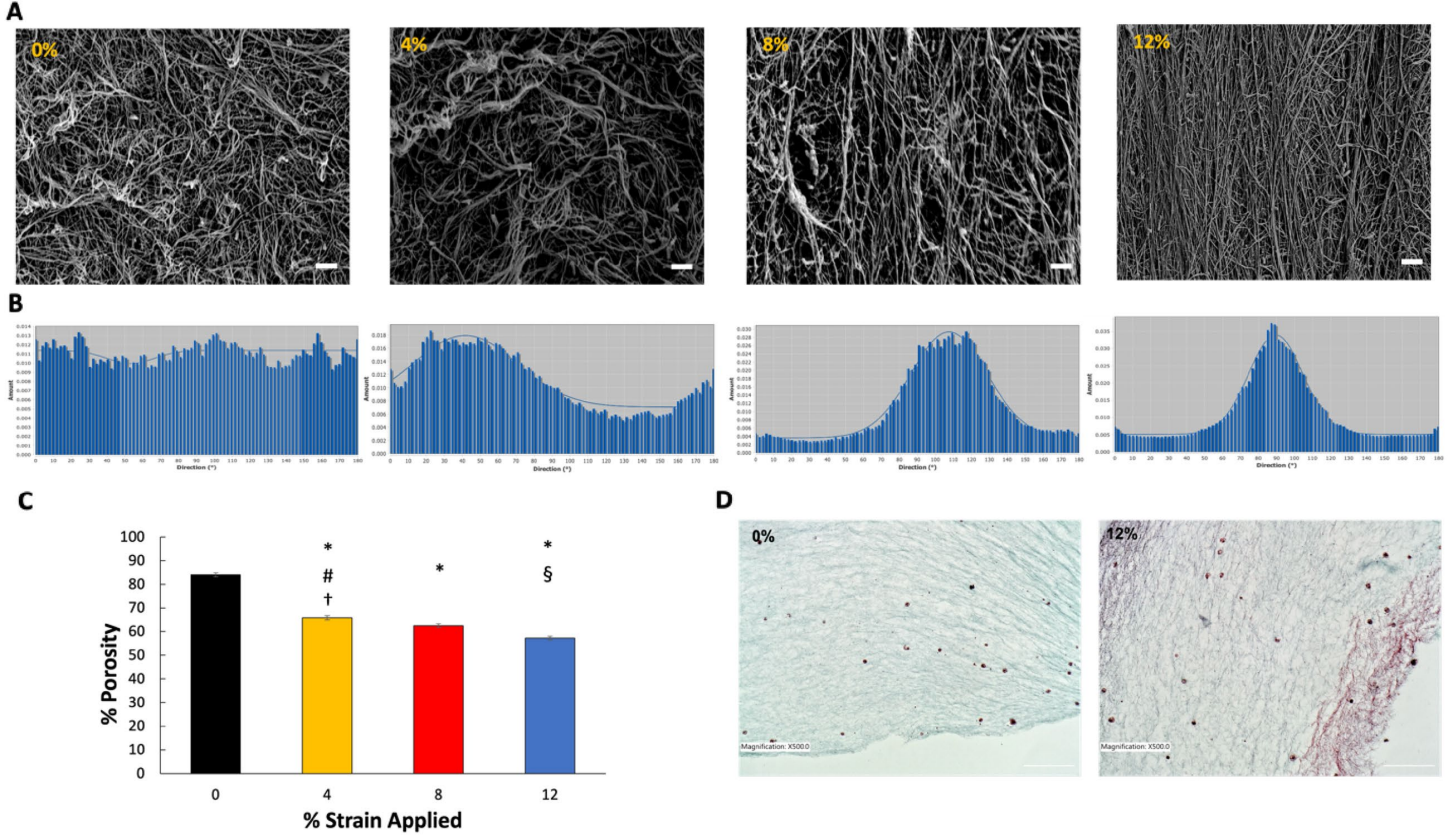
Effects of mechanical strain on fibrous protein remodeling and porosity within 3D tissue analogues. (**A**) SEM images of 0%, 4%, 8% and 12% mechanical strain 3D tissue analogues, scale bar represents 20 μm. (**B**) Directionality histograms of 3D tissue analogues subjected to 7 days of uniaxial loading, (**C**) %porosity graph, (**D**) H&E histological images of 0% and 12% mechanical strain 3D tissue analogues, scale bar represents 100 μm (n = 3). *Indicates significant difference with respect to 0% mechanical strain group (control). ^#^Represents a significant difference between 4 and 8% mechanical strain groups, ^§^represents a significant difference between 8 and 12% strain groups, and ^†^represents a significant difference between 4 and 12% strain groups, each with p < 0.05.

Mechanical stimulation had a profound effect on the extracellular fibrous protein (collagen and elastin) thicknesses and matrix porosity of the 3D tissue analogues. Figure 3A,D demonstrate the changes in matrix fibrous protein with the increased mechanical strain at the micro and tissue level using SEM and histology images, respectively. The SEM images of control (0%) and uniaxial tensile-loaded constructs at 0.1 Hz indicate that matrix organization is visible in the 3D analogues loaded at 8% and 12% uniaxial tensile strains, while control and 4% demonstrate random collagen fiber distribution. The SEM images (Fig. 3A) displayed denser de novo collagen fiber formations for the tissue exposed to 12% mechanical strain. As the percent strain increased, the fiber diameter started to increase, accompanied by the decrease in matrix porosity (Fig. 3C). While the porosity was 84.5 ± 0.23% for the control group, it was reduced to 57.4 ± 0.31% for the 12% mechanical strain group. Additionally, the alignment of collagen and elastin fibers increased as the mechanical strain increased. As seen in Fig. 3A, collagen and elastin fibers aligned parallel to the uniaxial tensile strain applied, with a higher alignment in the 12% group compared to all other groups. The histology images (Fig. 3D) further confirmed that matrix fiber density and thickness increased with the increased mechanical strain. The matrix thickness and fiber content increased 25 ± 0.9% in the 12% strain group compared to the counterparts within the control group. Comparing the SEM, histology images, and porosity results of the 0% and 12% strain groups show that the higher strain magnitude of 12% corresponding to the pathophysiological loading of musculoskeletal tissues depicts a significant increase in matrix organization and fibrous protein composition. These results demonstrate that such changes are most likely affected by mechanical stimulation-induced environments.

### Hyper-mechanical strain altered fibroblast morphology and fiber alignment within 3D tissue analogues

The matrix organization induced in the 3D tissue analogues due to the uniaxial tensile loading at different strains was visualized through SEM. The representative SEM images of samples loaded at 0%, 4%, 8%, and 12% at 0.1 Hz are shown in Fig. 3A. The SEM images of 0% (non-loaded) and loaded groups indicate that fiber alignment is clearly visible in the scaffolds loaded at 8% and 12% uniaxial tensile strains. In comparison, 0% demonstrate random collagen fiber distribution. To quantify the degree of fiber alignment and total matrix organization of each sample, directionality histograms were generated using Fiji/ImageJ. Figure 3B shows representative histograms for all mechanically loaded groups. The histogram data for the control group (0%) shows no definitive peak in the histogram, which indicates a random distribution of collagen/elastin fibers with a directionality amount of 0.012. With the increased applied mechanical strain to the cell-laden construct, the directionality increased significantly. While the directionality was 0.018 for the 4% strain group, it was 0.028 for 8% and 0.035 for the 12% mechanically loaded construct. Compared to the control group (0%), the directionality in the 12% group increased almost threefold. It is also observed that the peak in each loaded sample occurs at a definite angle, which implies that the fiber orientation of the tissue analogues is in the direction of the uniaxial tensile load application.

**Figure 4:**
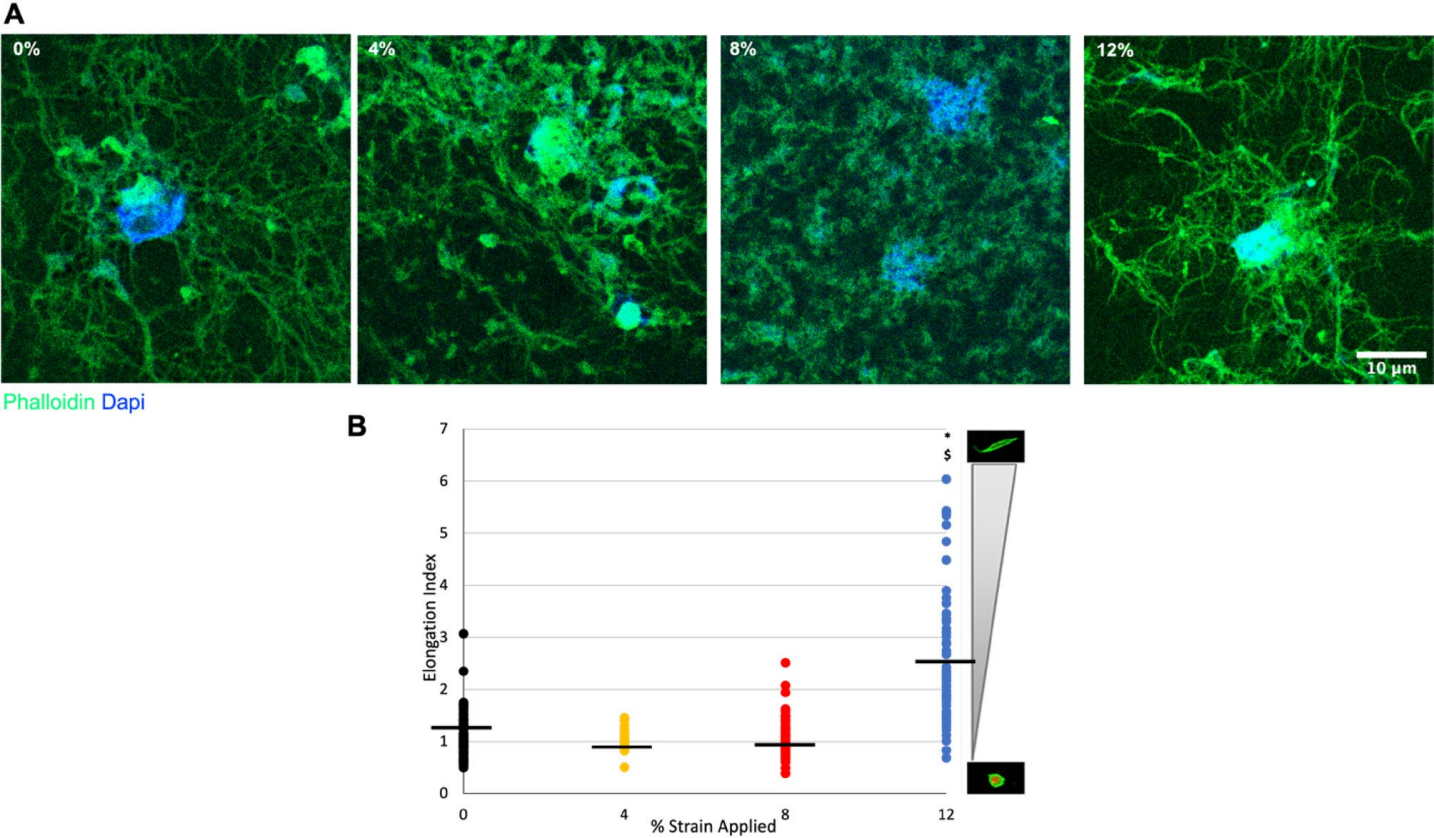
Cell elongation index (EI) analysis for HFF1 cells within the 3D tissue analogues after uniaxial loading. The gradient scale to the right designates the spectrum of cell measurements, with corresponding representative images of cell morphologies. *Indicates significant difference with respect to 0% mechanical strain group (control). ^#^Represents a significant difference between 4 and 8% mechanical strain groups, ^§^represents a significant difference between 8 and 12% strain groups, and ^†^represents a significant difference between 4 and 12% strain groups, each with P < 0.05.

To determine whether the changes in the extent of matrix directionality have any effects on the cells residing within the 3D tissue analogues, a cell elongation index (EI) was obtained from confocal images stained with F-actin (Phalloidin). Figure 4B demonstrates the cell EI for each group, where EI = 1 designates an amoeboid cell, and EI ≥ 2 is designated as an elongated cell. The black line represents the cell EI mean. Similarly, stained images (Fig. 4A) exhibit an amoeboid cell shape for 0%, 4%, and 8% uniaxially loaded groups, while the 12% strain loaded group exhibits a more cell elongated morphology with fibers extending in one direction out from the cell. Figure 4 demonstrates that the loaded samples at 12% strains show significant increases in residing cell morphology accompanied by a fiber alignment (Fig. 3A,B) compared to the control group.

### Hyper-mechanical strain upregulated profibrotic gene expressions for the cells within 3D tissue analogues

**Figure 5:**
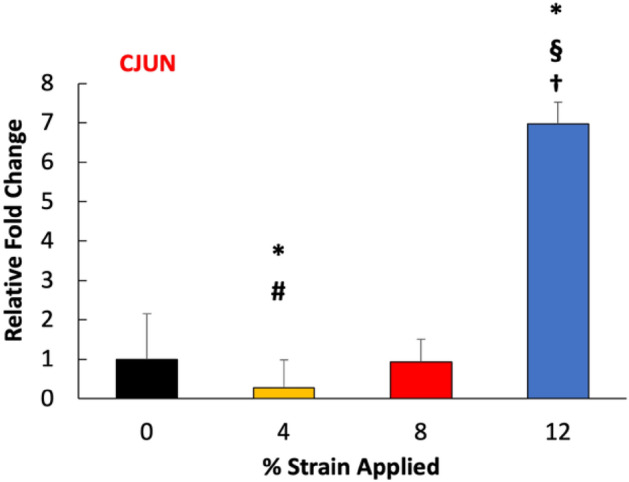
Effect of mechanical strain on the gene expression of early mechanoresponsive c-jun. *Indicate significant difference with respect to 0% strain group (control) with P < 0.05. ^#^Represent significant difference between 4 and 8% mechanical strain groups, ^§^represent a significant difference between 8 and 12% mechanical strain groups, and ^†^represent a significant difference between 4 and 12% strain groups, each with a 0.05 P-value.

The phenotypic changes of cells within the 3D tissue analogues upon various mechanical loading strains (0, 4, 8, and 12%) were assessed using gene expression analysis. Before conducting a thorough gene expression analysis, first, we studied whether mechanical strain applied to 3D tissue analogue would be translated into a cellular response. C-Jun is an immediate early mechanoresponsive gene expressed if mechanical perturbation is exerted on the tissue^39^. Following a 7-day mechanical loading, the c-jun expression increased almost sevenfold for the 12% strain group, and it was significantly different from the control (0%), 4, and 8% mechanical strain groups (Fig. 5).

**Figure 6:**
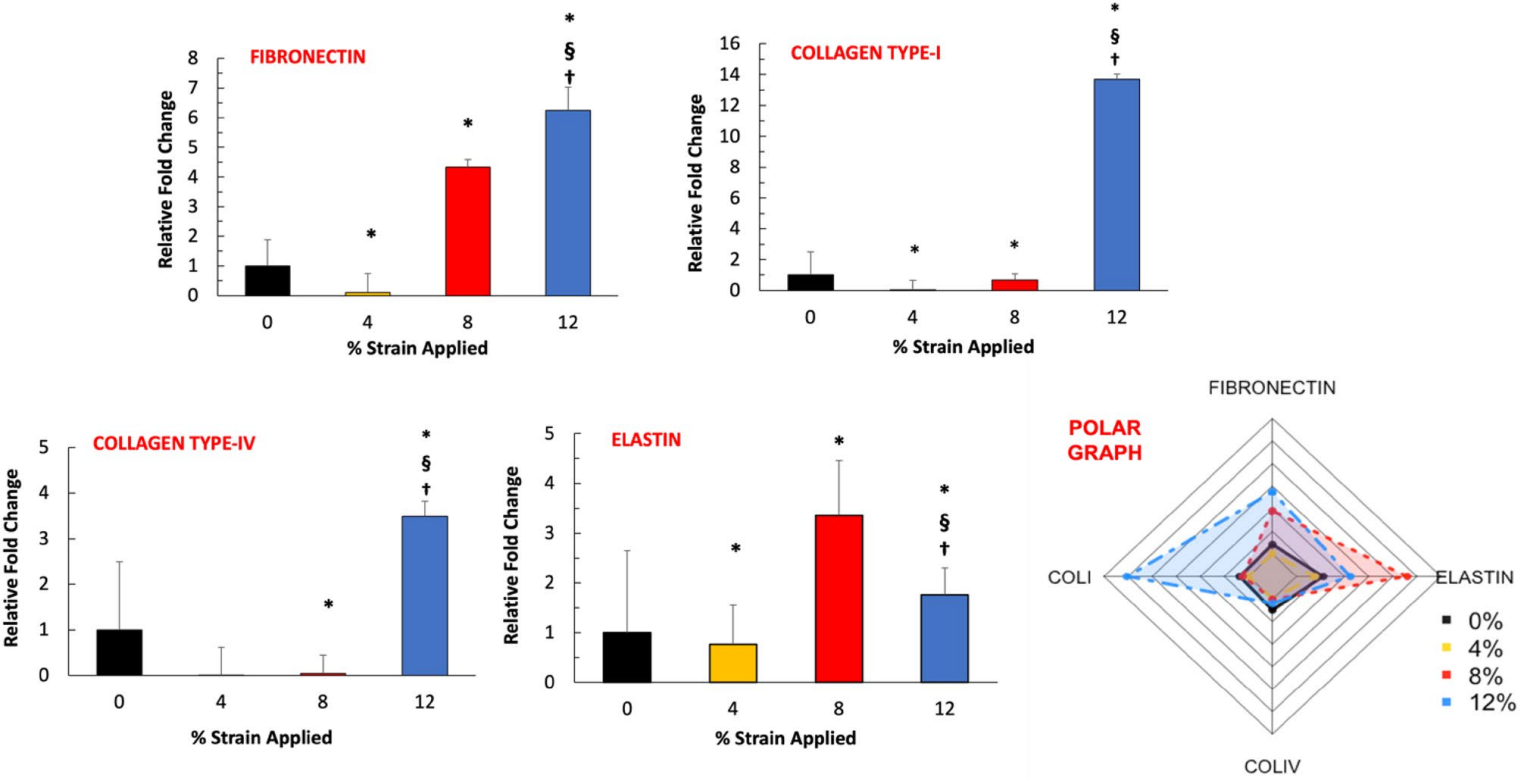
Effect of mechanical strain on the gene expression profiles of the native ECM markers. The polar graph illustrates the overall changes in ECM gene expression. *Indicate significant difference with respect to 0% strain group (control) with P < 0.05. ^§^Represent a significant difference between 8 and 12% mechanical strain groups, and ^†^represent a significant difference between 4 and 12% mechanical strain groups, each with a 0.05 P-value.

The increased expression of extracellular markers is a hallmark of fibrogenesis. Figure 6 demonstrates the relative fold change in mRNA expression of ECM fibrous proteins (collagen and elastin) and glycoprotein (fibronectin). The gene expression data demonstrated that expression of collagen type-IV, fibronectin, and collagen type-I increased with the increased applied mechanical strain amplitudes. A statistically significant increase in these markers was observed for cells exposed to 12% mechanical strain. The expression of collagen type-IV, fibronectin, and collagen type-I increased almost fourfold, sevenfold, and 13-fold, respectively, for the 12% mechanical strain group. The elastin expression demonstrated an increasing trend with increased mechanical strain until 12% mechanical strain. The elastin expression increased overall across all experimental groups; however, the highest expression was seen in the 8% group (threefold). In Fig. 6, the polar graph compiled and demonstrated the changes in expressions of ECM markers with the mechanical strain. The polar graph confirmed that collagen type-I and fibronectin expression increased substantially for 12% mechanical strain 3D tissue analogues.

**Figure 7:**
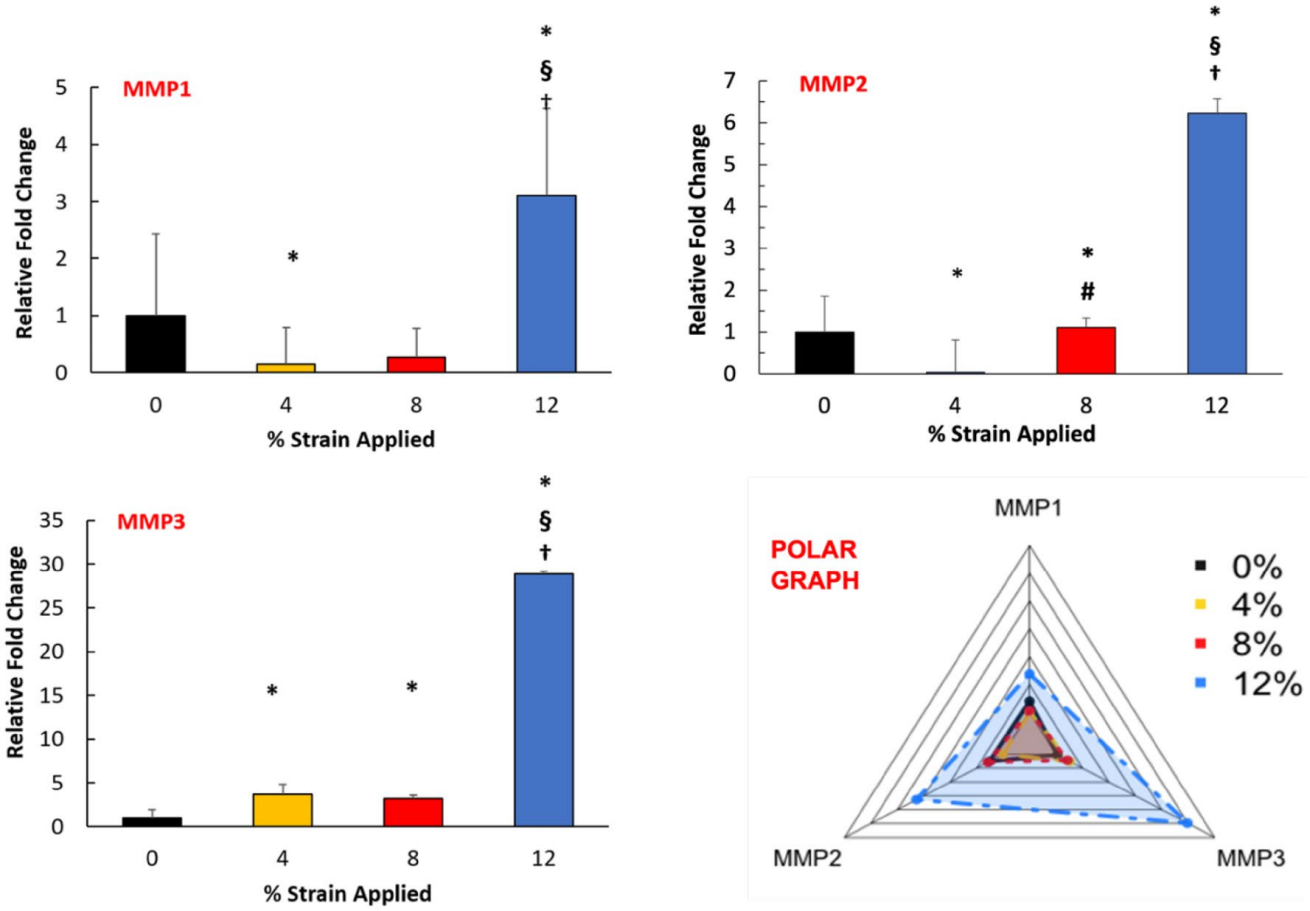
Effect of mechanical strain on the gene expression of MMPs. The polar graph illustrates the overall changes in MMPs gene expression. *Indicate significant difference with respect to 0% strain group (control) with P < 0.05. ^#^Represent significant difference between 4 and 8% strain groups, ^§^represent a significant difference between 8 and 12% mechanical strain groups, and ^†^represent a significant difference between 4 and 12% mechanical strain groups, each with a 0.05 P-value.

Since tissue fibrosis results from dysregulation between the synthesis and degradation of ECM molecules^40^, the expression of important matrix metalloproteinases (MMPs) was also assessed upon mechanical strain application. Figure 7 demonstrates the changes in MMP 1,2, and 3 gene expressions with varying mechanical strain amplitudes. The data indicated a trend of increased MMPs expression following increased mechanical strain stimulation. The increment was substantially significant for 12% mechanical strain 3D tissue analogue since MMP1, MMP2, and MMP3 expressions elevated around threefold, sixfold, and 30-fold, respectively.

**Figure 8:**
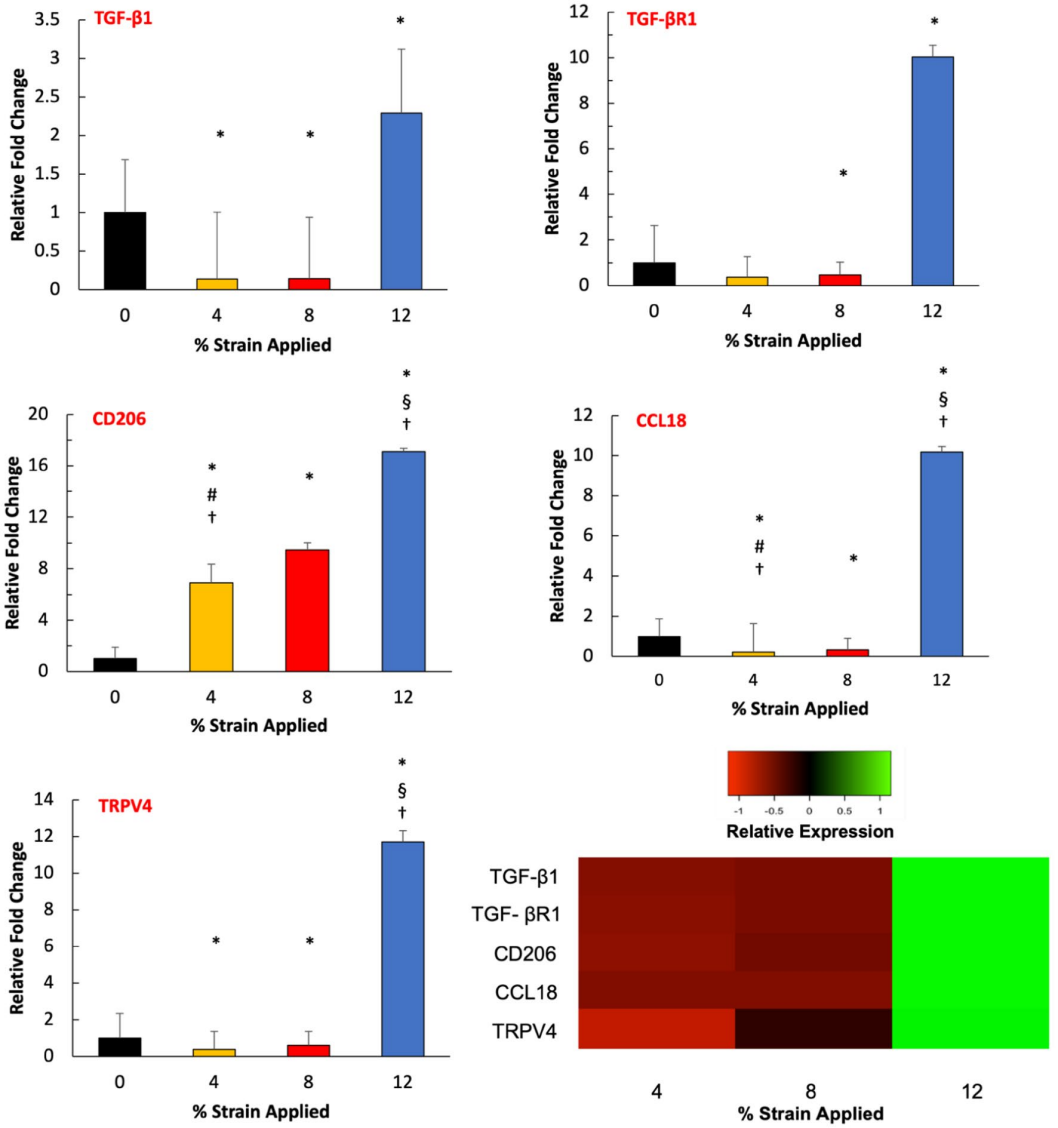
Effect of mechanical strain on the gene expression of activation stressors markers and profibrotic markers within 3D tissue analogues. The heatmap represents a color-coding of average z-scores obtained from mRNA fold changes. Green indicates an expression greater than the mean; red indicates an expression lower than the mean, and black indicates a quantity close to the mean (n = 3). *Indicate significant difference with respect to 0% mechanical strain group (control) with P < 0.05. ^#^Represent significant difference between 4 and 8% mechanical strain groups, ^§^represent a significant difference between 8 and 12% mechanical strain groups, and ^†^represent a significant difference between 4 and 12% strain groups, each with a 0.05 P-value.

Following assessing ECM markers and MMPs, the expressions of fibroblast activation stressors and profibrotic cytokine, and chemokines were analyzed as a function of mechanical strain amplitudes (Fig. 8). The expression of fibroblast activation stressors, TGF-β1 and TGF-βR1, increased almost 2.5-fold and 11-fold for the 12% mechanical strain group compared to control groups, respectively. On the other hand, TGF-β1 expression decreased significantly for the 4% and 8% mechanical strain groups. The profibrotic cytokine and chemokines expressions of CD206, CCL18, and TRPV4 demonstrated a similar trend. The CD206 is upregulated 16-fold in the 12% mechanical strain group, while CCL18 and TRPV4’s expressions increased tenfold and 12-fold, respectively. For fibroblast activation stressors and profibrotic markers, their expressions are significantly (P < 0.05) higher in 12% mechanical strain groups compared to the counterparts in the 4% and 8% mechanical strain groups.

### Hyper-mechanical strain-induced FMT within 3D tissue analogues

**Figure 9:**
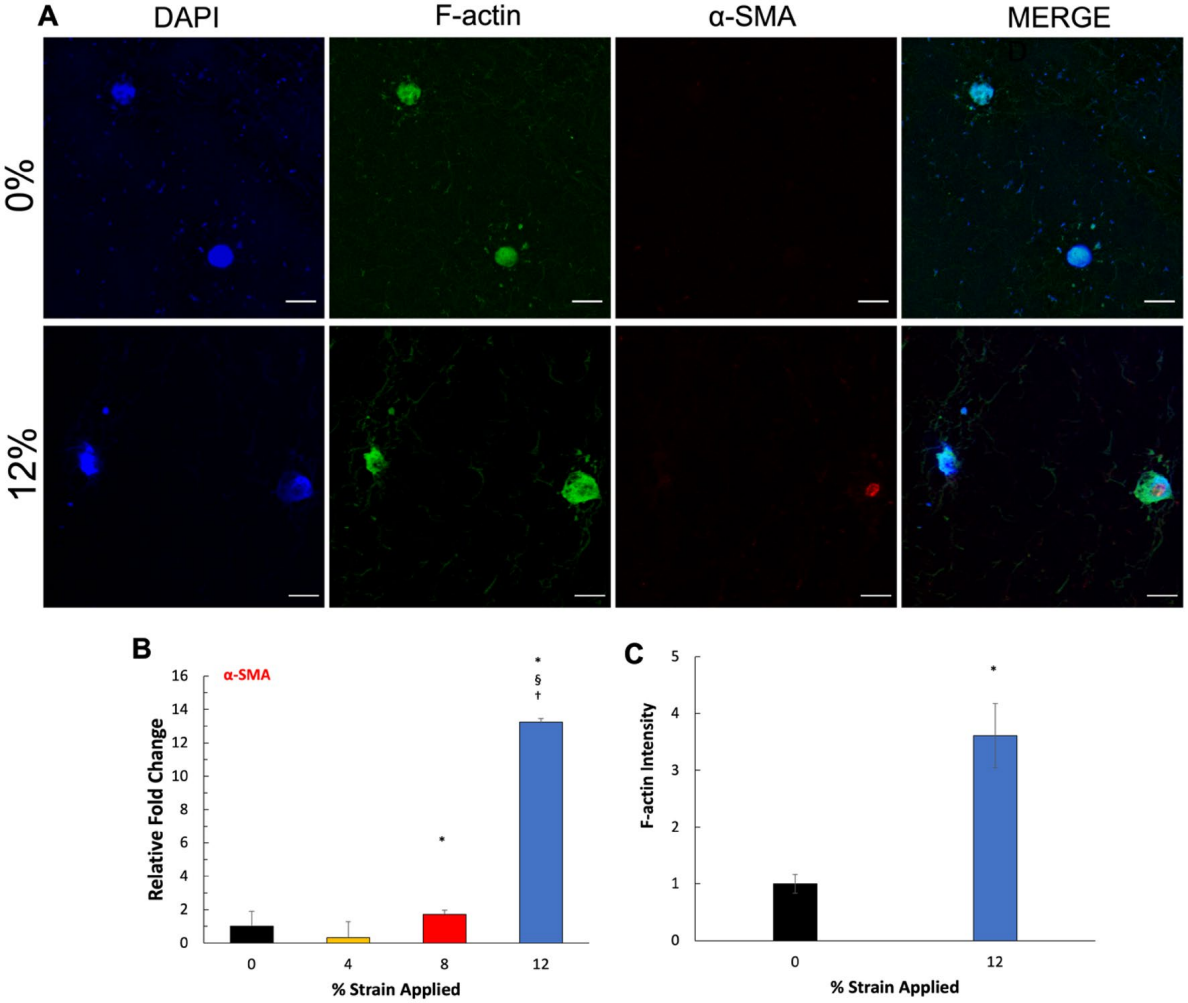
Mechanical strain induces FMT in 3D tissue analogues. (**A**) α-SMA IHC staining of 0% (control) and 12% strain group, (**B**) relative gene expression of α-SMA, (**C**) F-actin immunostaining (green), and nucleus staining (blue) within 3D tissue analogues. Scale bar represents 10 μm. (**C**) Relative F-actin fluorescence intensity within 3D tissue analogues. *Indicate significant difference with respect to 0% mechanical strain group (control) with P < 0.05. ^§^Represent a significant difference between 8 and 12% mechanical strain groups, and ^†^represent a significant difference between 4 and 12% mechanical strain groups, each with a 0.05 P-value.

After confirming that fibroblast activation stressors and profibrotic markers expressions were upregulated with the increased mechanical strain amplitude, the full fibroblast-to-myofibroblast transition was confirmed by analyzing the myofibroblast marker for α-smooth muscle actin (α-SMA). α-SMA is one of the most prominent and widely accepted markers for fully differentiated myofibroblasts^41^. Figure 9B demonstrates the changes in α-SMA mRNA expression as a function of applied mechanical strain. The α-SMA expression elevated significantly (P < 0.05) for 8% and 12% mechanical strain groups by 1.7 ± 0.3 and 13.2 ± 0.2-folds, respectively, compared to the control group (0% mechanical strain). On the other hand, no α-SMA expression was detected for the 4% mechanical strain group. To visualize the changes in pro-fibrotic markers’ expressions as a function of mechanical loading, the gene expression data was translated into a heatmap graph in which markers that were upregulated were highlighted in green, and downregulated were highlighted in red. The compiled gene expression data in the heatmap graph further demonstrated that fibrogenesis within 3D tissue analogue initiated with higher mechanical strain applications. While some of the profibrotic markers started to express relatively low in the 8% mechanical strain group, all hallmark fibrogenesis markers, including fibroblast activation stressors, pro-fibrotic markers, and myofibroblast markers, upregulated significantly for the 12% mechanical strain group (Fig. 8). To further confirm the effects of mechanical loading on FMT behavior, IHC staining (Fig. 9A) of α-SMA on 0% and 12% strain groups were obtained. Figure 9A showed the expression of α-SMA in the 12% strain group, while 0% (control) did not show any expression of α-SMA at the protein level. Another important myofibroblast marker is the formation of filamentous F-actin stress fibers, which can provide a measure of myofibroblast activation^42^. To this end, we stained for F-actin (Fig. 9A) and calculated the intensity relative to control (0% mechanical strain) within the 3D tissue analogue (Fig. 9C). Tissue analogues exposed to 12% strain showed a 3.6-fold increase in F-actin expression.

## Discussion

It is accepted that mechanical loading plays an integral role in musculoskeletal tissue regeneration and rehabilitation. There is an overwhelming amount of evidence suggesting that the mechanical environment around the musculoskeletal tissues affects the homeostasis, regeneration, and disease state of the tissues. In the absence of mechanical loading, musculoskeletal tissues lose their strength, modulus, toughness, and collagen alignment^43–53^. While insufficient mechanical loading creates stress-shielding for musculoskeletal tissues, the hyper mechanical loading causes deterioration of tissue structure and promotes degeneration^54–56^. Thus, in this study, we investigated how changes in mechanical strain amplitudes applied to cell-laden 3D tissue analogue modulated tissue regeneration and fibroblast-to-myofibroblast transition, which is an indicator fibrotic pathway.

Quantification of tissue cellularity demonstrated an increase in the cell number with the higher mechanical strain amplitudes (Fig. 2). 3D tissue analogues subjected to 12% mechanical strain demonstrated an almost 2.5-fold increase in cells over 7 days of stimulation compared to the non-loaded samples (0% strain). The spatial growth of cells in relation to the mechanical strain was increased. As shown in Fig. 2A, the cell morphology in the 12% group changed and aligned parallel to the direction of the uniaxial tensile strain applied, while cells in the unstimulated group (control) demonstrated a more rounded shape, with no changes in its morphology. These changes in cell morphology, shape, and elongation follow the expected cell behavior under FMT^57–59^. There was a slight increase in the cell numbers for the 4% and 8% mechanical strain groups; however, it was not significantly different from the control group (0%) (Fig. 2C). The increase in the total number of cells shown in the 12% group is solely due to cell proliferation since the cell viability across all mechanically stimulated groups, and the control group is statistically the same (Fig. 2B). The substantial increase in cellularity for 12% mechanical strain tissue was accompanied by an increase in de novo collagen deposition, as confirmed with SEM and histology images (Fig. 3A,D). Both SEM and histology images and their analyses further suggested that the porosity of 3D analogue decreased with increased mechanical strain amplitudes (Fig. 3C) which can be attributed to densely synthesized collagen fibers.

The cell morphology demonstrated significant changes under hyper mechanical strain (12%) condition. The cells within 0%, 4% and 8% loaded matrix demonstrated more spherical-like morphology (Fig. 4A) with similar elongation index values (Fig. 4B). Yet, the fibroblast residing in the 12% mechanical strain group demonstrated notably elongated cell structure with the presence of cytoplasmic extensions along the collagen fibers. The cells within the 12% group were reoriented within the matrix as the matrix itself aligned with the increased mechanical loading (Fig. 3A,B). SEM images of loaded tissue analogues showed increased collagen fiber alignment with the increased strain, while the control group demonstrated a highly random matrix organization (Fig. 3A). Our previous studies have shown that the degree of matrix organization and fiber alignment can vary significantly based on the type of cells encapsulated and the stimulation regime^30,32,60^. As shown in Figs. 2 and 3, higher cell densities within the tissue analogues increase the extent of matrix arrangement and increase the diameter of collagen fibers. Thus, this matrix organization and fiber alignment is attributed to the combinational effect of uniaxial tensile loads and cell proliferation within the analogues.

While the structural changes were prominent towards fibrotic tissue formation with increased mechanical strain amplitudes, the changes in cellular level were also important to assess the FMT. The changes in c-jun expression were assessed as a function of mechanical strain amplitudes because the elevated expression of c-jun indicates that cells residing within the tissue sense the external mechanical stimuli and trigger mechanical strain-activated responses^61,62^. The c-jun gene expression result (Fig. 5) demonstrated a significant increase in its expression for the residing cells within the tissue exposed to 12% mechanical strain compared to counterparts in control groups (0% mechanical strain). This suggested that the higher mechanical strain amplitude applied to the cell-laden 3D tissue analogue was effectively transferred to the cellular level and initiated the molecular changes. For lower mechanical strain amplitudes (4 and 8% strain), there was not a significant increase in c-jun expression, which may suggest that longer mechanical stimulation time and/or higher frequency were required to translate the applied mechanical loading into the cell with similar efficiency as in 12% mechanical strain.

The expressions of specific matrix proteins are also a major regulator of fibroblast-to-myofibroblast transition^63^. In this study, we assessed how fibrous ECM markers and glycol ECM markers change with the various mechanical strain amplitudes. The cells within the 3D tissue exposed to 12% mechanical strain displayed a significantly greater ability to express fibrous ECM markers (collagen type-I, collagen type IV, elastin) along with glycol ECM markers like fibronectin (Fig. 6). This data suggested that the higher mechanical strain (12%) induced hypercellularity and was accompanied by an increase in de novo synthesis of ECM markers, both of which are the early signs of fibroblast transition from the homeostatic state to activated (or proto-myofibroblast) state. Our data agree with the literature since prominent studies also demonstrated that increased mechanical stimulation promotes native ECM protein expressions and tissue secretion. This overexpression of ECM proteins is often seen in the development of fibroproliferative diseases such as keloid, cardiovascular fibrosis, glomerulosclerosis, and idiopathic pulmonary fibrosis^64–66^. For instance, Booth et al.^67^ demonstrated that the ECM composition was completely different in idiopathic pulmonary fibrosis (IDF) compared to normal lung tissue. Several ECM proteins including collagen type-I and collagen type IV only expressed in the IDF tissue but not in healthy tissue. Overexpression of ECM markers further changes the cell activity and further promotes fibrosis progression^68^.

Upon initiation of FMT, there is a dysregulation of tissue remodeling machinery which results in an excessive ECM deposition^69^. To overcome the ECM accumulation, the cells start to express MMPs to degrade superimposed collagens, elastin, and proteoglycans within the ECM^14^. Thus, in this study, to understand whether overexpressed ECM markers due to increased mechanical loading affect the MMPs expressions, we measured the expressions of MMPs 1, 2 and 3 as a function of mechanical strain amplitudes (Fig. 7). Our data demonstrated a statistically significant increase in the expression of MMPs 1, 2, and 3, following the trend of increased mechanical strain amplitudes. The highest intensification in MMP expression was observed for the 12% mechanical strain group with almost threefold, 6.5-fold, and 30-fold changes compared to the control group for MMPs 1, 2, and 3, respectively. While the MMP expressions were also increased for 4 and 8% mechanical strain groups compared to the control group, they were significantly less than the 12% mechanical strain group. These data can be attributed to the orchestrated expression of MMPs and ECM proteins in homeostatic tissue. The increased expression of MMPs (Fig. 7) and ECM (Fig. 6) suggest that there is an imbalance in matrix turnover, which is another significant hallmark of FMT leading to fibrotic tissue formation. MMPs primarily digest the ECM proteins and fibers; however, they also trigger the signaling pathways responsible for secreting bioactive mediators, including growth factors, cytokines, chemokines, and cell-surface receptors^70,71^. In fibrogenesis, several MMPs, including MMP1 and 3, are up-regulated, which subsequently promote a vicious cycle of excessive ECM deposition and hyper scar tissue formation^70^.

The cellular and structural characterization data (Figs. 2, 3, 4) so far indicated the initiation of proto-myofibroblast phenotype and myofibroblast differentiation from the homeostatic fibroblasts under various mechanical loading amplitudes. To confirm the pro-fibrotic differentiation of fibroblasts, the expression of activation stressors and pro-fibrotic markers were assessed. The relative gene expression data (Fig. 8) demonstrated that expressions of the fibroblast activation stressors, TGF-β1, and its receptor, TGF-βR1, increased 2.5-fold and tenfold, respectively, for the 12% mechanical strain group compared to control groups. This suggested that hyper-mechanical loading initiated the first crucial step of the fibrotic process: proto-myofibroblasts differentiation. It is well-established that fibrosis is initiated with the activation of fibroblasts through activation stressors, including TGF-β1 and its receptor TGF-βR1^72,73^. Following the stressor-induced activation of fibroblasts, these can secrete profibrotic cytokines and chemokines, further promoting myofibroblast differentiation^13,74^. The gene expression results in Fig. 8 agreed with the literature since upregulation of TGF-β1 and TGF-βR1 further promoted the expression of the profibrotic markers, including mannose receptor (CD206), CCL18, and transient vanilloid receptor type-4 (TRPV4). Numerous in vitro and in vivo studies demonstrated that CD206 and CCL18, a well-established anti-inflammatory cytokine and chemokine, promote fibroblast transition to myofibroblasts and collagen synthesis, resulting in fibrosis^75,76^. In addition, TRPV4 involves increased cellularity, ECM synthesis, and fibroblast to myofibroblast differentiation^76^. The TRPV4 expressions are upregulated in the later stage of myocardial, pulmonary, and skin fibrosis^77^. The heatmap in Fig. 8 demonstrated that with higher mechanical strain (12%) the expressions of profibrotic markers are upregulated with the increased stress activators expression, which confirmed the complete fibroblast to myofibroblast transformation.

The fibroblast-to-myofibroblast transition is described as a multifactorial process that involves enhanced force generation with enlarged focal adhesions and incorporation of α-SMA into stress fibers^78,79^. The myofibroblast differentiation is accompanied by upregulated α-smooth muscle actin (α-SMA) and a formation of pronounced actin stress fibers^80^. While the proto-myofibroblasts exist in healthy tissue structures such as a wall of pulmonary alveoli, they do not express α-SMA. In contrast, well-developed or fully differentiated myofibroblasts are rich in α-SMA and are observed in fibrotic lesions^81^. The α-SMA gene expression results (Fig. 9B) show that, compared to the control group (0% mechanical strain), α-SMA expression increased significantly (P < 0.05) for 8% and 12% mechanical strain groups with almost twofold change and 13-fold, change respectively. Furthermore, Fig. 9A confirmed the protein expression of a-SMA in the 12% compared to the 0% (control). No expression of a-SMA at the protein level was seen in any other group but the 12% mechanically stimulated group. In particular, the cells exposed to hyper-mechanical strain (12%) demonstrated a dramatic increase in α-SMA expression. However, increased α-SMA could be due to the increase in soluble α-SMA monomers (G-actin) without being incorporated into stress fibers^82–85^. Thus, we immuno-stained the filamentous α-SMA monomers (F-actin) to understand whether the dramatically increased α-SMA expression in the 12% strain group was due to the increase in filament formation. F-actin immunostaining and its quantification confirmed higher levels of F-actin in the 12% mechanical strain group compared to the control (0%), with an almost 25% increase (Fig. 9C). This result further confirmed the full myofibroblast differentiation under high mechanical strain exposure since F-actin does express in low-level in fibroblast in the homeostatic stage and starts to be visible during proto-myofibroblast differentiation^42^.

In summary, the mechanical loading exerted on fibroblast-laden 3D tissue analogue can initiate autocrine and paracrine tissue healing via mechanical strain amplitude-dependent manner. The encapsulated fibroblast demonstrated proliferative, contractile, and matrix synthetic states in response to increased mechanical strain amplitude. Supplementary Figure 1, provided as a Supplementary Document, demonstrated the schematic representation of the results. While up to 8% mechanical strain (uniaxial tensile load) applied to the tissue promoted tissue regeneration without triggering the stress activators and fibroblast-to-myofibroblast transition (FMT). The mechanical strain beyond 8% in particular 12% mechanical strain, pushed the fibroblast into full myofibroblast differentiation with pronounced α-SMA expression and stress fiber formation. For tissue function in a mechanically active tissue environment in which the tissue and residing cells are exposed to myriad mechanical strains, a better understanding of FMT and associated changes in cellular and tissue levels are crucial for targeted approaches to halting fibrosis and hypertrophic scarring.

## Supplementary Information

Supplementary Information.

## Data Availability

The datasets generated during the current study are not publicly available due to the pending patent application but are available from the corresponding author on reasonable request.
