# Supplementary material for: Mechanoresponsive regulation of fibroblast-to-myofibroblast transition in three-dimensional tissue analogues: mechanical strain amplitude dependency of fibrosis
Source: Sci Rep. 2022 Oct 7;12:16832. doi: 10.1038/s41598-022-20383-5 (PMC9547073; doi:10.1038/s41598-022-20383-5)
Supplement: Supplementary file 1 — Supplementary Information. [file 41598_2022_20383_MOESM1_ESM.docx]

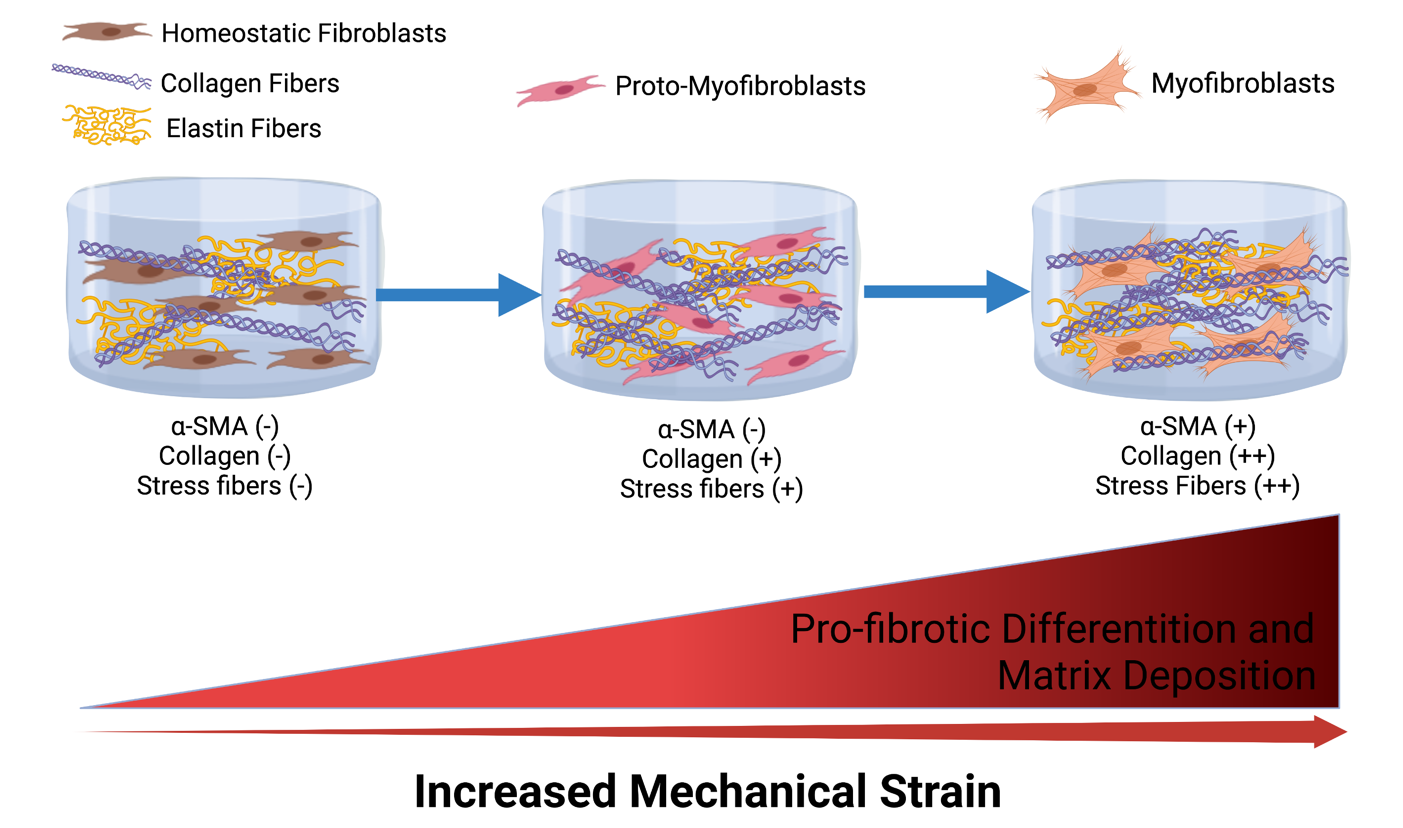


**Supplementary Figure 1.** Relationship between the degree of mechanical strain applied on the tissue and fibroblast-to-myofibroblast transition. Increased mechanical strain leads to the formation of proto-myofibroblast and subsequent differentiation to myofibroblasts. “Created with Biorender.com”

**Table 1.** Forward and reverse primers for real-time PCR

| **Gene** | **Forward Primer** | **Reverse Primer** | **Ref.** |
| --- | --- | --- | --- |
| MMP-1 | 5’-CAACTTACATCGTGTTGCGG-3’ | 5’-ACCGGACTTCATCTCTGTCG-3’ | (34) |
| MMP-2 | 5’-CCAAGAACTTCCGTCTGTCC-3’ | 5’-TGAACCGGTCCTTGAAGAAG-3’ | (34) |
| MMP-3 | 5’-CAGCCAACTGTGATCCTGCT-3’ | 5’-CTTCATATGCGGCATCCACG-3’ | (34) |
| ELN | 5’-GGTGTAGGTGGAGCTTTTGC-3’ | 5’-CTGTTGGGTAACCAGCCTTG-3’ | (35) |
| FBN | 5’-GTTATGCTGGTTGTACAGGG-3’ | 5’-CATCATCGTAACACGTTGCC-3’ | (35) |
| COL I | 5’-GTTGGTGCTAAGGGTGAAGC-3’ | 5’-GTTGGTGCTAAGGGTGAAGC-3’ | (35) |
| COL IV | 5’-TTCGACTTGCGGCTCAAAG-3’ | 5’-CCACGCTCTCCTTTCAATCC-3’ | (35) |
| CJUN | 5’-TTCTCTCCGTCGCAACTTGT-3’ | 5’-GCCAACTCATGCTAACGCAG-3’ | (36) |
| TRPV4 | 5’-ACCATCCTGGACATTGSGGG-3’ | 5’-CACCTCATCCACCCTGAAGC-3’ | (37) |
| CD206 | 5’-CTACAAGGGATCGGGTTTATGGA-3’ | 5’-TTGGCATTGCCTAGTAGCGTA-3’ | (38) |
| CCL18 | 5’-AAGAGCTCTGCTGCCTCGTCTA-3’ | 5’-CCCTCAGGCATTCAGCTTAC-3’ | (38) |
| α-SMA | 5’-GTCAGCACTTCGCATCAAGG-3’ | 5’-TTCACAGGATTCTGGGAGCGG-3’ | (35) |
| TGF-β1 | 5’-GGTTATCTTTTGATGTCACCG-3’ | 5’-GTTATGCTGGTTGTACAGGG-3’ | (35) |
| TGFR-β1 | 5’-GTGACAGATGGGCTCTGCTT-3’ | 5’-AGCAATGGTAAACCAGTAGTTGG-3’ | (35) |
| GAPDH | 5’-AGAAGGCTGGGGCTCATTTG-3’ | 5’-AGGGGCCATCCACAGTCTTC-3’ | (38) |
